# The HIF‐1α pathway plays a critical role in salivary gland development in *ex vivo* organ cultures

**DOI:** 10.1002/2211-5463.13351

**Published:** 2021-12-24

**Authors:** Tomomasa Kimura, Manabu Sakai, Nao Gojo, Mikio Watanabe, Narikazu Uzawa, Takayoshi Sakai

**Affiliations:** ^1^ Department of Oral‐facial Disorders Osaka University Graduate School of Dentistry Suita Japan; ^2^ Department of Oral and Maxillofacial Surgery 2 Osaka University Graduate School of Dentistry Suita Japan; ^3^ Department of Clinical Laboratory Osaka University Dental Hospital Suita Japan; ^4^ Department of Clinical Laboratory and Biomedical Sciences Osaka University Graduate School of Medicine Suita Japan

**Keywords:** development, HIF‐1α, hypoxia, salivary glands

## Abstract

The transcription factor, hypoxia‐inducible factor‐1α (HIF‐1α), has previously been shown to upregulate the expression of hypoxia‐related genes, including erythropoietin (EPO). However, the role of hypoxia‐inducible factor‐1α in morphogenesis during salivary gland development is unclear. We investigated the function of HIF‐1α in submandibular gland (SMG) organ cultures obtained from embryonic day 13.5 embryos from ICR female mice. Expression of HIF‐1α, glucose transporter 1, and vascular endothelial growth factor was induced under hypoxia (5% O_2_). We further showed that BAY 87‐2243‐mediated inhibition of HIF‐1α suppressed salivary gland development. Under severe hypoxia (1% O_2_), HIF‐1α did not promote salivary gland development; this was due to suppression of cell proliferation and inhibition of the cell cycle and not because of autophagy and apoptosis. Additionally, using the inhibitor U0126, we verified that the ERK1/2 pathway is upstream of HIF‐1α. Overall, we found that the HIF‐1α signaling pathway plays a critical role in salivary gland development in *ex vivo* SMG organ cultures.

## Graphical abstract

The role of the HIF‐1α signaling pathway in salivary gland development is currently unknown. Through the use of inhibitors, including U0126 and BAY87‐2243, we demonstrated that the HIF‐1α signaling pathway, including phosphorylated ERK and HIF‐1α signaling pathway‐related proteins (VEGF and GLUT1), plays a critical role in salivary gland development in *ex vivo* organ culture.

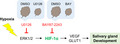

**Abbreviations**
GLUT1: glucose transporter 1
HIF‐1α: hypoxia‐inducible factor‐1α
HRE: hypoxia‐response element
IGF‐2: insulin‐like growth factor‐2
PCNA: proliferating cell nuclear antigen
P‐ERK1/2: phosphorylated ERK1/2
PHD: prolyl hydroxylation domain protein
pVHL: von Hippel–Lindau tumor suppressor
SMG: submandibular salivary gland
VEGF: vascular endothelial growth factor

In a hypoxic environment, erythropoietin (EPO), a hematopoietic promoting factor, is produced to increase the number of red blood cells so that oxygen can be transported efficiently [1]. In 1995, hypoxia‐inducible factor‐1 (HIF‐1) was identified as a transcription factor that binds to the hypoxia response element (HRE) under hypoxic conditions and upregulates EPO gene expression [2, 3]. HIF is a heterodimeric complex consisting of a hypoxia‐inducible subunit, HIF‐α (isoforms HIF‐1α, HIF‐2α, and HIF‐3α), and the constitutively expressed, HIF‐1β. Under normoxic (20% O_2_) conditions, specific prolyl residues of HIF‐α are hydroxylated by prolyl hydroxylation domain protein (PHD). These residues are then recognized by the von Hippel–Lindau tumor suppressor (pVHL), which mediates ubiquitination of HIF‐α, hence targeting it to proteasomal degradation HIF, which is activated by hypoxia, a transcription factor consisting of a heterodimer of α and β subunits. HIF activated by hypoxia is a transcription factor consisting of heterodimers of subunits and β‐subunit alpha [4]. This results in the inactivation of the HIF‐mediated hypoxic response. Under hypoxic conditions, PHD activity is suppressed, and HIF‐1α is not hydroxylated and thus protected from proteasomal degradation. Consequently, HIF‐1α stabilizes and translocates into the nucleus to form a heterodimer with HIF‐β and binds to the HRE. As a result, HIF‐mediated hypoxic response is activated. Hypoxia induces the transcription of genes involved in various cell functions, such as erythropoietin, vascular endothelial growth factor (VEGF), glucose transporter 1 (GLUT1), and insulin‐like growth factor 2 (IGF‐2) [2, 3]. HIF hematopoiesis, angiogenesis, cellular energy metabolism, cell proliferation, and cell death control various biological events, such as autophagy.

Salivary glands secrete approximately 1–1.5 L of saliva daily. Saliva has digestive, antibacterial, mucosal protective, and bolus‐forming properties and hence plays an important role in maintaining the environment and function of the oral cavity and the body as a whole [5]. During the development of some organs including salivary glands, a branching process which begins on embryonic day 12 (E12) is necessary [6, 7, 8]. Early organs are simple epithelial buds that gradually develop into polycystic structures. Bud branching continues during early fetal development and peaks between E13 and E15. We previously used *ex vivo* submandibular salivary gland (SMG) organ cultures to observe SMG branching morphogenesis between E13 and E15 [7, 8]. Many researchers have used our method to investigate the interactions between cells and matrices, cell proliferation, and cell death. Various factors are involved in salivary gland morphogenesis during embryonic development [8, 9, 10]. Embryos are exposed to low oxygen levels during development, and hence, HIF‐1α plays an important role in embryonic development [11, 12]. Organs, including salivary glands, develop under hypoxic conditions. However, the role of HIF‐1α in the development of salivary glands is poorly understood. In this study, we analyzed the function of HIF‐1α in salivary gland development using *ex vivo* SMG organ cultures.

## Materials and methods

### Organ culture

All animal experiments were carried out in accordance with the recommendations of the Guide for the Care and Use Committee of Osaka University Graduate School of Dentistry, Osaka, Japan. The protocol was approved by the Committee on the Ethics of Animal Experiments of Osaka University Graduate School of Dentistry (permit number: 30‐024‐0). All surgeries were performed under a combination of three anesthetics: 0.3 mg·kg^−1^ medetomidine hydrochloride (Nippon Zenyaku Kogyo, Fukushima, Japan), 4 mg·kg^−1^ midazolam (Astellas Pharma, Tokyo, Japan), and 5 mg·kg^−1^ butorphanol tartrate (Meiji Seika Pharma, Tokyo, Japan). All efforts were made to minimize animal suffering and to use minimal number of animals. Female ICR mice were purchased from Japan SLC, Inc. (Hamamatsu, Japan), and housed in cages (one animal per cage) containing wood fiber bedding with food and water supplied *ad libitum* under a 12 h light–dark cycle at 23 °C. E13.5 SMGs were isolated from female ICR mice. First, the mandibles were separated from the embryo by segregating the lower mandible and removing the tissue surrounding the tongue and oral epithelium using forceps on a glass dissection plate. Under a stemi‐2000CS stereo microscope (Carl Zeiss, Jena, Germany), the mandible was placed onto a glass dissection plate, and each SMG was isolated from the tongue. The selected SMGs were placed on Nucleopore membranes (1 µm pore size, GE Healthcare UK Ltd, Buckinghamshire, England) in serum‐free Dulbecco’s modified Eagle’s medium/F12 medium (Thermo Fisher Scientific, Waltham, MA, USA) and cultured at 37 °C, as previously described [6, 13].

### Western blot analysis

The SMGs were lysed with RIPA buffer (Nacalai Tesque, Kyoto, Japan) supplemented with protease and phosphatase inhibitors (Nacalai Tesque). Cell lysates were centrifuged (15,000 r.p.m.) for 20 min, and the supernatants were heated at 95 °C for 5 min in denaturing Laemmli buffer (Bio‐Rad Laboratories Inc., Hercules, CA, USA). Proteins were separated by SDS/PAGE and transferred onto polyvinylidene difluoride (PVDF) membranes (Bio‐Rad Laboratories Inc.). The membranes were blocked with 3% low‐fat milk in Tris‐buffered saline containing Tween 20 and then incubated with anti‐HIF‐1α (1 : 1000, Cell Signaling Technology, Beverly, MA, USA), anti‐GLUT1 (1 : 10,000, Abcam, Cambridge, MA, USA), anti‐VEGF (1 : 100, Invitrogen, Carlsbad, CA, USA), anti‐ERK1/2 (1 : 1000, Cell Signaling Technology), anti‐P‐ERK1/2 (1 : 1000, Cell Signaling Technology), anti‐LC3B I/II (1 : 1000, Cell Signaling Technology), anti‐caspase‐3 (1 : 1000, Cell Signaling Technology), anti‐Procaspase‐3 (1 : 1000, Cell Signaling Technology), anti‐PCNA (1 : 5000, BD, San Diego, CA, USA), anti‐Cyclin D1 (1 : 10,000, Abcam, Cambridge, MA, USA), or anti‐actin (1 : 1000, Sigma Aldrich, Saint Louis, MO, USA). The bound antibodies were detected with anti‐rabbit IgG, HRP‐linked antibody (1 : 1000, Cell Signaling Technology), or anti‐mouse IgG, HRP‐linked antibody (1 : 1000, Cell Signaling Technology), using the ECL detection kit (Bio‐Rad Laboratories Inc.).

### Inhibitor treatment

Signal transduction inhibitors, BAY 87‐2243 (HIF‐1α inhibitor, Selleck Chemicals, Houston, TX, USA), U0126 (ERK1/2 inhibitor, Cell Signaling Technology), and DMOG (PDH inhibitor, Sigma‐Aldrich) were resuspended in DMSO (Wako, Tokyo, Japan) and added to serum‐free medium at the indicated concentrations. BAY 87‐2243 was used at concentrations of 50 and 100 μm, U0126 at 0, 10, 20, and 40 μm concentration, and DMOG at 0 and 2000 μm concentration. The control medium contained the same volume of DMSO. SMGs were treated with BAY 87‐2243 and U0126 for 4 h and cultured for an additional 4 or 30 h under hypoxic conditions. SMGs that were treated with U0126 were cultured for a further 4 or 30 h under normoxic conditions as a control group. SMGs that were treated with DMOG were cultured for 10 h. For immunoblotting, SMGs were collected 4 h after hypoxia treatment. Photographs were taken at 0 and 30 h after hypoxia treatment using a digital SLR camera (Fuji FinePix, Fuji, Tokyo, Japan) fitted onto an Axiovert 40C phase‐contrast microscope (Carl Zeiss).

### Hypoxia treatment

E13.5 SMGs cultured for 18 h were exposed to hypoxic conditions (5% or 1% O_2_, in 5% CO_2_ at 37 °C) for 4 h or 30 h using a BIONIX‐1 hypoxic culture kit (Sugiyama‐Gen Co., Ltd., Tokyo, Japan), which consisted of a gas concentration regulating agent (Sugiyama‐Gen), an OXY‐1 oxygen monitor (JIKCO, Tokyo, Japan), a plastic bag (Mitsubishi Gas Chemical, Tokyo, Japan), and clips to close the bag.

### Statistical analysis

Student’s *t*‐test was used to determine the *P*‐value for statistical significance. Bonferroni’s multiple comparison post *t*‐test was combined with a one‐way analysis of variance (ANOVA) to compare differences between the means within an experiment. Statistical significance was set at *P* < 0.05. Results expressed as mean ± standard error (SE) values.

## Results

### HIF‐1α is expressed under 5% oxygen conditions

**Fig. 1:**
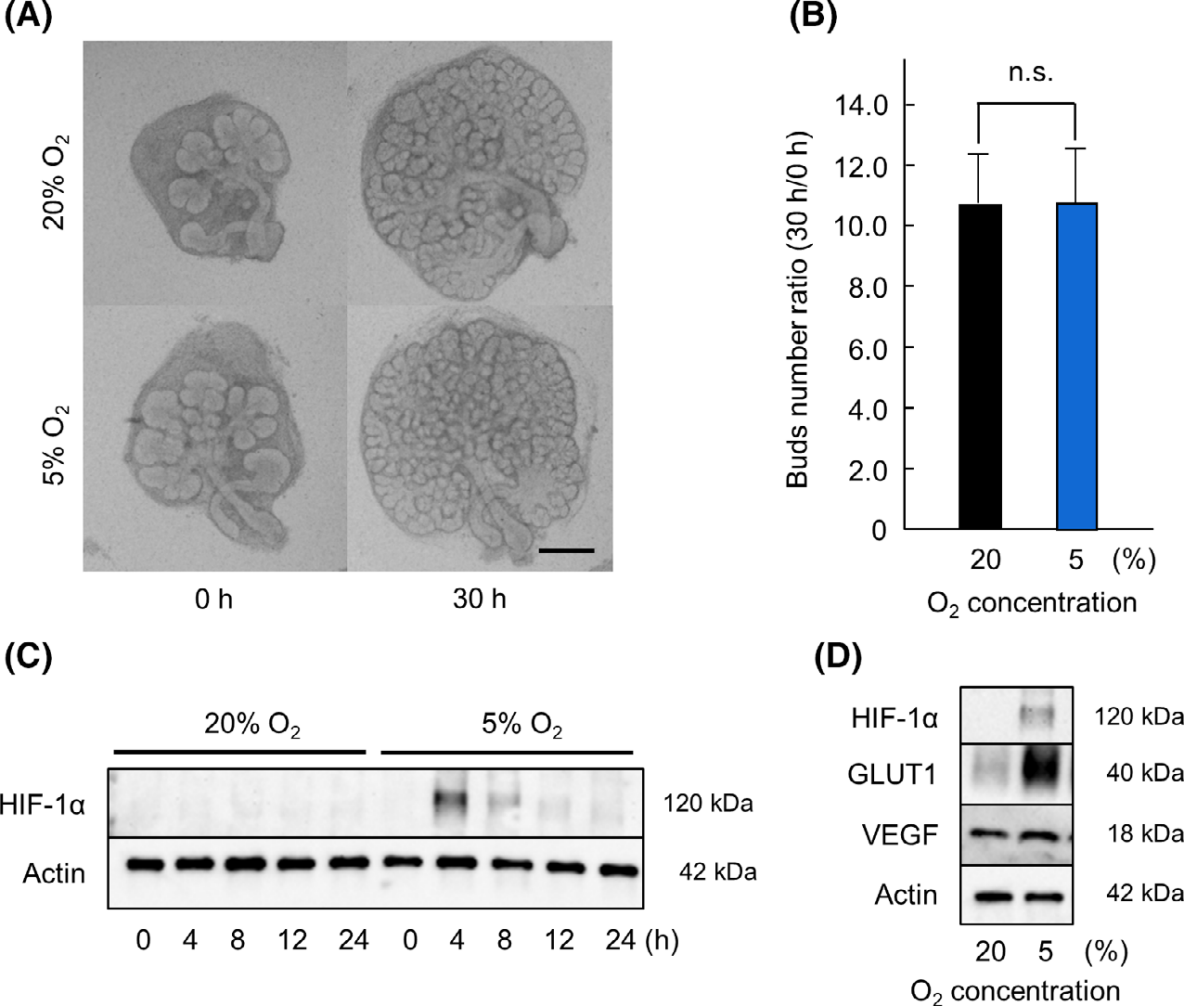
SMG branching morphogenesis in *ex vivo* organ culture under normoxic and hypoxic conditions. Phase‐contrast images show E13.5 + 18 h cultured SMGs after 0 and 30 h of culture with DMSO under normoxic (20% O_2_) and hypoxic (5% O_2_) conditions (*n* = 6) (A). The effect of hypoxic (5% O_2_) conditions was quantified by counting the number of buds per gland at 0 and 30 h (*n* = 6) (B). The expression of HIF‐1α in E13.5 + 18 h cultured SMGs after 4, 8, 12, and 24 h of culture with DMSO under normoxic (20% O_2_) and hypoxic (5% O_2_) conditions was analyzed by western blot analysis (C). The expression of HIF‐1α, GLUT‐1, VEGF, and actin in E13.5 + 18 h cultured SMGs after 4 h of culture with DMSO under normoxic (20% O_2_) and hypoxic (5% O_2_) conditions were analyzed by western blot analysis (D). Bars represent the mean ± SEM. n.s. indicates not significant (*P* > 0.05) (Student’s *t*‐test). Scale: 250 μm.

Previous reports have shown that HIF‐1α is involved in the development of the fetal stage and hypoxic conditions [11, 12]. The role of HIF‐1α in salivary gland development has not been evaluated in *ex vivo* organ cultures. In this study, we investigated the expression of HIF‐1α during salivary gland development. We first cultured embryonic SMG under normoxic (20% O_2_) and hypoxic (5% O_2_) conditions to observe the effect on morphology and the number of branching buds. The number of branching buds in the SMG culture is a measure of salivary gland development and was determined at 0 and 30 h. We found no differences in SMG morphology and the number of buds (Fig. 1A, B). Therefore, we speculated that HIF‐1α may contribute to salivary gland development under hypoxic conditions. The SMG culture was treated under normoxic (20% O_2_) and hypoxic (5% O_2_) conditions for 0, 4, 8, 12, and 24 h. Western blot analysis was performed to detect the expression of HIF‐1α. HIF‐1α was not expressed under normoxic conditions but was expressed under hypoxic conditions (Fig. 1C). Since HIF‐1α expression was observed after 4 h exposure to hypoxic conditions, we considered this a suitable time point for western blot analysis. GLUT1 and VEGF are downstream of HIF‐1α [2]. GLUT1 is involved in the glucose uptake required to maintain respiration in all cell types [14]. VEGF plays an important role in angiogenesis and lymphangiogenesis in both physiological and pathological conditions [15]. We examined the expression of GLUT1 and VEGF in SMG cultured under hypoxic (5% O_2_) conditions and normoxic (20% O_2_) conditions (Fig. 1D). The expression of GLUT1 and VEGF verifies that HIF‐1α is expressed under hypoxic conditions and suggests that it may be involved in salivary gland development.

### BAY 87‐2243 suppresses SMG development via inhibition of HIF‐1α

**Fig. 2:**
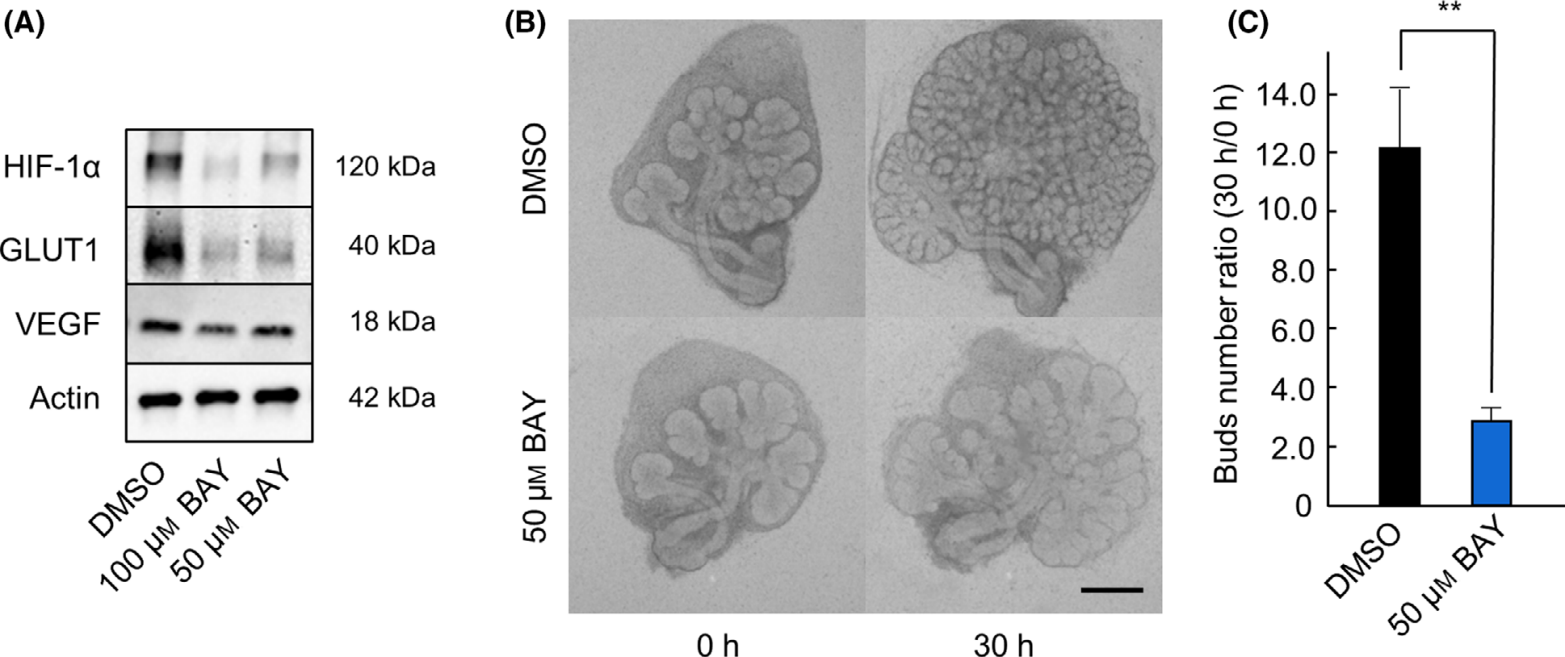
Effect of BAY 87‐2243 on SMG development under hypoxic condition. The expression of HIF‐1α, GLUT1, and VEGF in E13.5 + 18 h cultured SMGs after 4 h of culture with DMSO, 50 μm BAY 87‐2243, and 100 μm BAY 87‐2243 was analyzed by western blot analysis (A). Phase‐contrast images show E13.5 + 18 h cultured SMGs after 0 and 30 h of culture with DMSO and 50 μm BAY 87‐2243 (*n* = 6) (B). The effect of BAY 87‐2243 treatment was quantified by counting the number of buds per gland at 0 and 30 h (*n* = 6) (C). Bars represent the mean ± SEM. **P < 0.01 compared with DMSO (Student’s *t*‐test). Scale: 250 μm.

To determine whether HIF‐1α is involved in salivary gland development, we performed experiments using the HIF‐1α inhibitor BAY 87‐2243 [16, 17]. First, we investigated whether BAY 87‐2243 (50, 100 μm) inhibited the expression of HIF‐1α, GLUT1, and VEGF under hypoxic (5% O_2_) conditions by western blot analysis. We found that BAY 87‐2243 reduced the expression of HIF‐1α, GLUT1, and VEGF in a concentration‐dependent manner (Fig. 2A). Next, we observed the morphology of BAY 87‐2243‐treated SMG under hypoxic (5% O_2_) conditions. BAY 87‐2243‐treated SMG exhibited smaller size as well as decreased bud number compared to DMSO‐treated SMG (Vehicle Control) (Fig. 2B, C). These results indicate that inhibition of HIF‐1α by BAY 87‐2243 suppressed salivary gland development.

### U0126 suppresses SMG development via inhibition of HIF‐1α

**Fig. 3:**
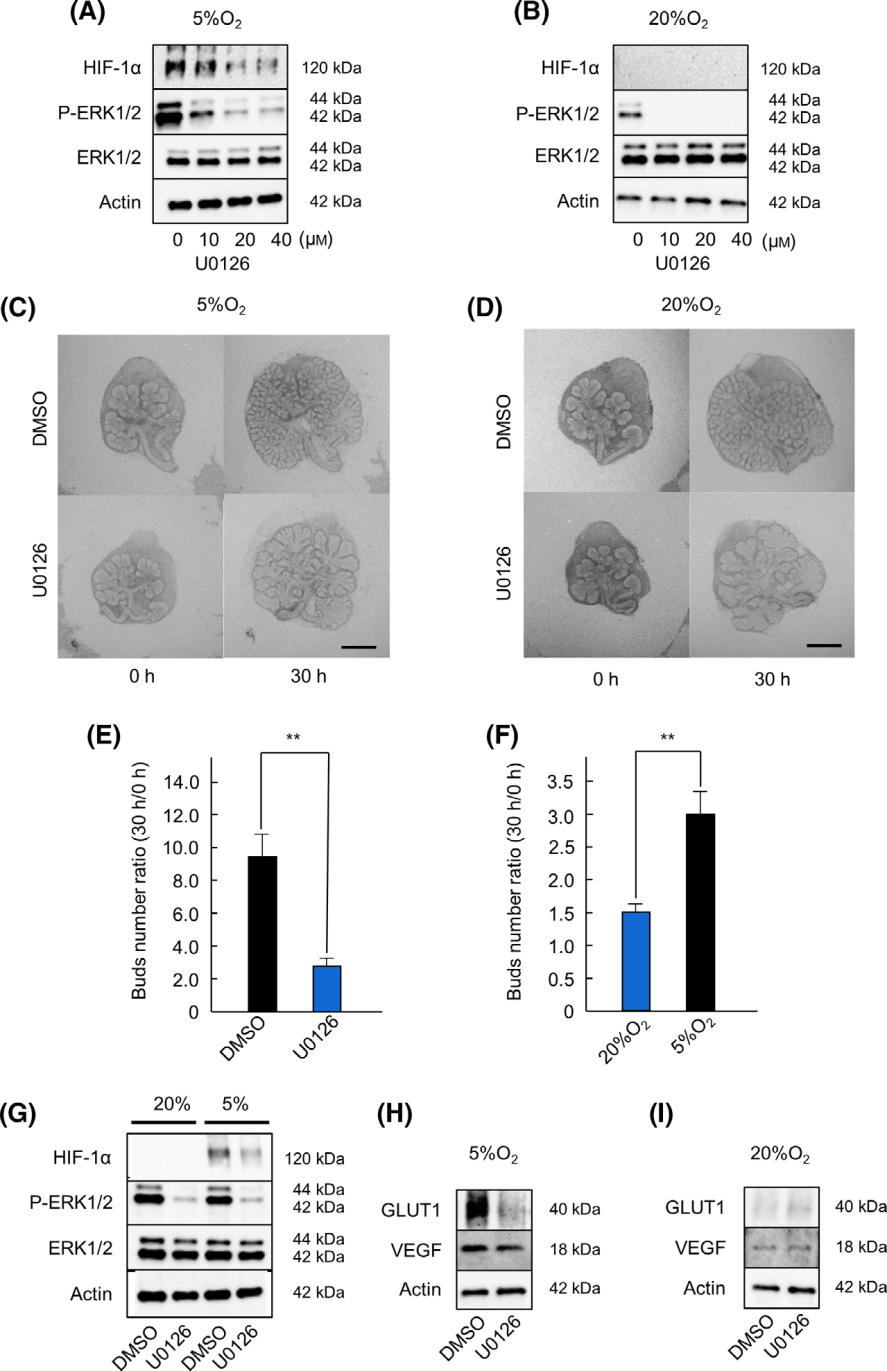
Effect of U0126 on SMG development and phosphorylation of ERK1/2 under hypoxic condition. The expression of HIF‐1α, phosphorylated‐ERK1/2, and ERK1/2 in E13.5 + 18 h cultured SMGs after 4 h of culture under hypoxic (5% O_2_) and normoxic (20% O_2_) conditions with 0, 10, 20, and 40 μm U0126 were analyzed by western blot analysis (A, B). Phase‐contrast images show E13.5 + 18 h cultured SMGs after 0 and 30 h of culture under hypoxic (5% O_2_) and normoxic (20% O_2_) conditions with DMSO and 20 μm U0126 (*n* = 6) (C, D). The effect of U0126 treatment was quantified by counting the number of buds per gland at 0 and 30 h (*n* = 6) (E). The effect of U0126 treatment under normoxic (20% O_2_) and hypoxic (5% O_2_) conditions was compared by counting the number of buds per gland at 0 and 30 h (*n* = 6) (F). The expression of HIF‐1α, phosphorylated‐ERK1/2, and ERK1/2 in E13.5 + 18 h cultured SMGs after 4 h of culture with DMSO and U0126 under normoxic (20% O_2_) and hypoxic (5% O_2_) conditions was analyzed by western blot analysis (G). The expression of GLUT1 and VEGF in E13.5 + 18 h cultured SMGs after 4 h of culture under hypoxic (5% O_2_) and normoxic (20% O_2_) conditions with DMSO and U0126 was analyzed by western blot analysis (H, I). Bars represent the mean ± SEM. **P < 0.01 compared with DMSO (Student’s *t*‐test). Scale: 250 μm.

Previous studies have shown that HIF‐1α is downstream of the ERK1/2 signaling pathway [18]. The ERK1/2 inhibitor U0126 has been used to investigate the function of HIF‐1α signaling [19, 20]. Therefore, we used U0126 (0, 10, 20, 40 μm) to analyze the role of HIF‐1α‐related pathways in salivary gland development. We performed western blot analysis to detect the expression of HIF‐1α and p‐ERK1/2 in U0126‐treated SMG under hypoxic (5% O_2_) conditions. Normoxic (20% O_2_) conditions were used as a control for western blot analysis. We found that U0126 inhibited the expression of HIF‐1α and p‐ERK1/2 in a concentration‐dependent manner (Fig. 3A,B). Next, we observed the morphology of SMG on treatment with U0126 under hypoxic (5% O_2_) and normoxic (20% O_2_) conditions. U0126‐treated SMG showed moderate branching and epithelial endbud elongation (Fig. 3C,D). We also counted the number of buds after 30 h treatment with 20 μm U0126, under both conditions. U0126 treatment considerably decreased the number of buds (Fig. 3E). Comparing the two conditions, the number of buds observed under normoxic conditions was approximately 50% of that observed under hypoxic conditions (Fig. 3F). Next, we compared the expression of HIF‐1α, GLUT1, VEGF, and p‐ERK1/2 by western blot analysis in U0126‐treated SMG under normoxic (20% O_2_) and hypoxic (5% O_2_) conditions. We found that U0126 reduced the expression of GLUT1 and VEGF only under hypoxic (5% O_2_) conditions (Fig. 3G,H,I). Additionally, ERK1/2 increased the expression of GLUT1 and VEGF via HIF‐1α under hypoxic conditions. Taken together, these results indicate that ERK1/2 is upstream of HIF‐1α and is involved in salivary gland development under hypoxic conditions.

### U0126 and BAY 87‐2243 affected cell proliferation, cell cycle, and apoptosis

**Fig. 4:**
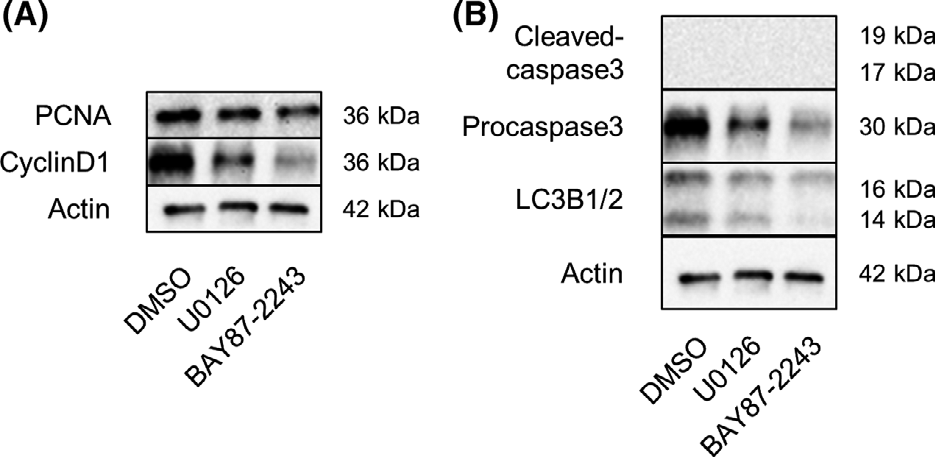
U0126 and BAY 87‐2243 affected cell proliferation, cell cycle, and apoptosis. The expression of PCNA, Cyclin D1, Cleaved‐caspase‐3, Procaspase‐3, LC3B1/2, and actin in E13.5 + 18 h cultured SMGs after 4 h of culture with DMSO, U0126, or BAY 87‐2243 under hypoxic (5% O_2_) condition was analyzed by western blot analysis (A, B)

To determine whether U0126 and BAY 87‐2243 affect cell proliferation and cell cycle, we performed western blot analysis of proliferating cell nuclear antigen (PCNA), Cyclin D1, Cleaved‐caspase‐3, Procaspase‐3, and LC3B1/2. We observed that both U0126 and BAY 87‐2243 decreased the expression of PCNA, Cyclin D1, and Procaspase‐3 (Fig. 4A,B). The expression of LC3B1/2 was also affected by BAY 87‐2243 treatment. These results indicate that U0126 and BAY 87‐2243 affect cell proliferation, cell cycle, and apoptosis.

### Expression of HIF‐1α in hypoxia is important for SMG development

**Fig. 5:**
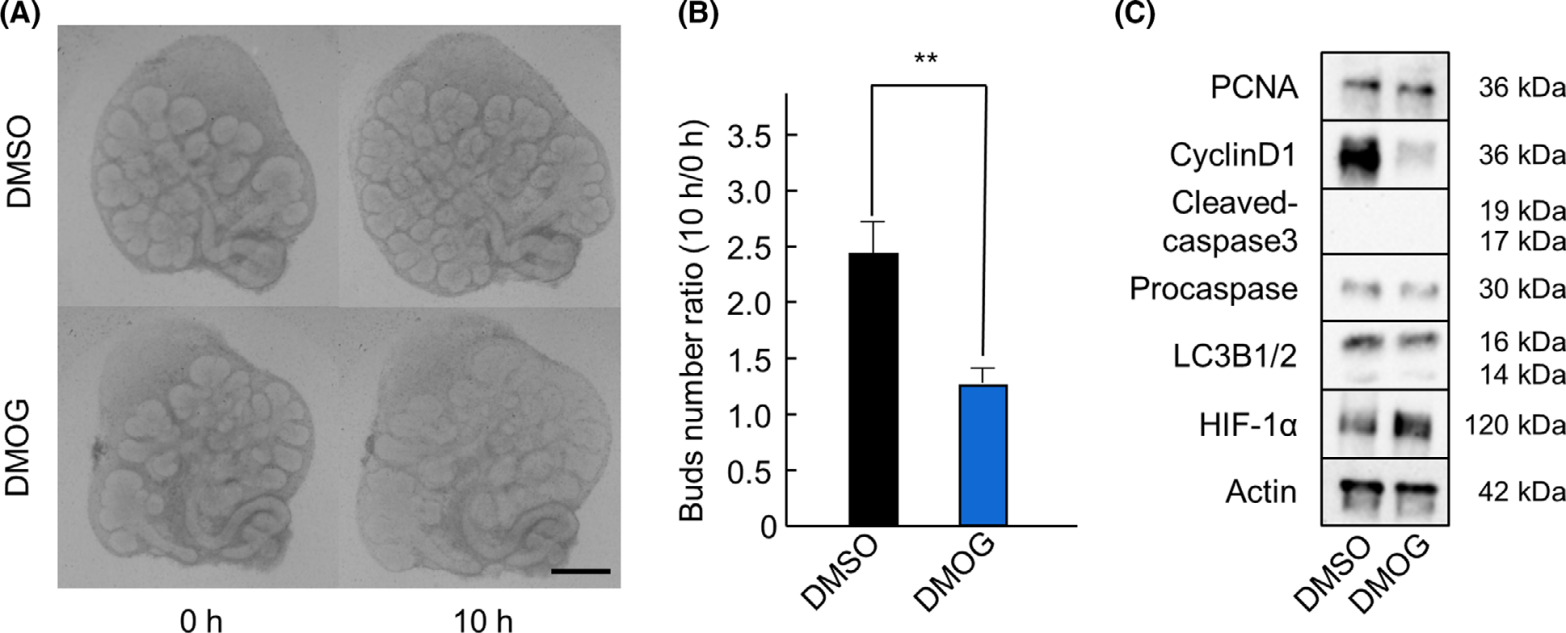
Expression of HIF‐1α in hypoxia is important for SMG development. Phase‐contrast images show E13.5 + 18 h cultured SMGs after 0 and 10 h of culture with 2000 μm of DMOG under normoxic (20% O_2_) condition (*n* = 6) (A). The effect of DMOG under normoxic condition was quantified by counting the number of buds per gland at 0 and 10 h (*n* = 6) (B). The expression of PCNA, Cyclin D1, Cleaved‐caspase‐3, Procaspase‐3, LC3B1/2, and actin in E13.5 + 18 h cultured SMGs after 4 h of culture with DMSO or 2000 μm of DMOG under normoxic (20% O_2_) and hypoxic (5% O_2_) conditions was analyzed by western blot analysis (C). Scale: 250 μm.

To investigate the effect of HIF‐1α stabilization on SMG development under normoxia (20% O_2_), we cultured SMGs in the presence of 2000 μm DMOG, a PHD inhibitor. First, we investigated the growth of SMGs cultured under normoxia for 10 h, in the presence of DMOG. The results showed that the bud size increased, while the number of buds decreased (Fig. 5A,B). We then performed western blot analysis. The expression of HIF‐1α increased in DMOG treatment. Interestingly, the expression of Cyclin D1 was higher under hypoxia (5% O_2_), despite lower HIF‐1α expression. There was however no difference in cell proliferation, apoptosis, and autophagy, between the groups (Fig. 5C). These results suggest that the expression of HIF‐1α under hypoxic conditions is important for cell cycle activation and that cell cycle cannot be promoted by drug‐induced HIF‐1α stabilization under normoxia.

### HIF‐1α is expressed under severe hypoxic conditions

**Fig. 6:**
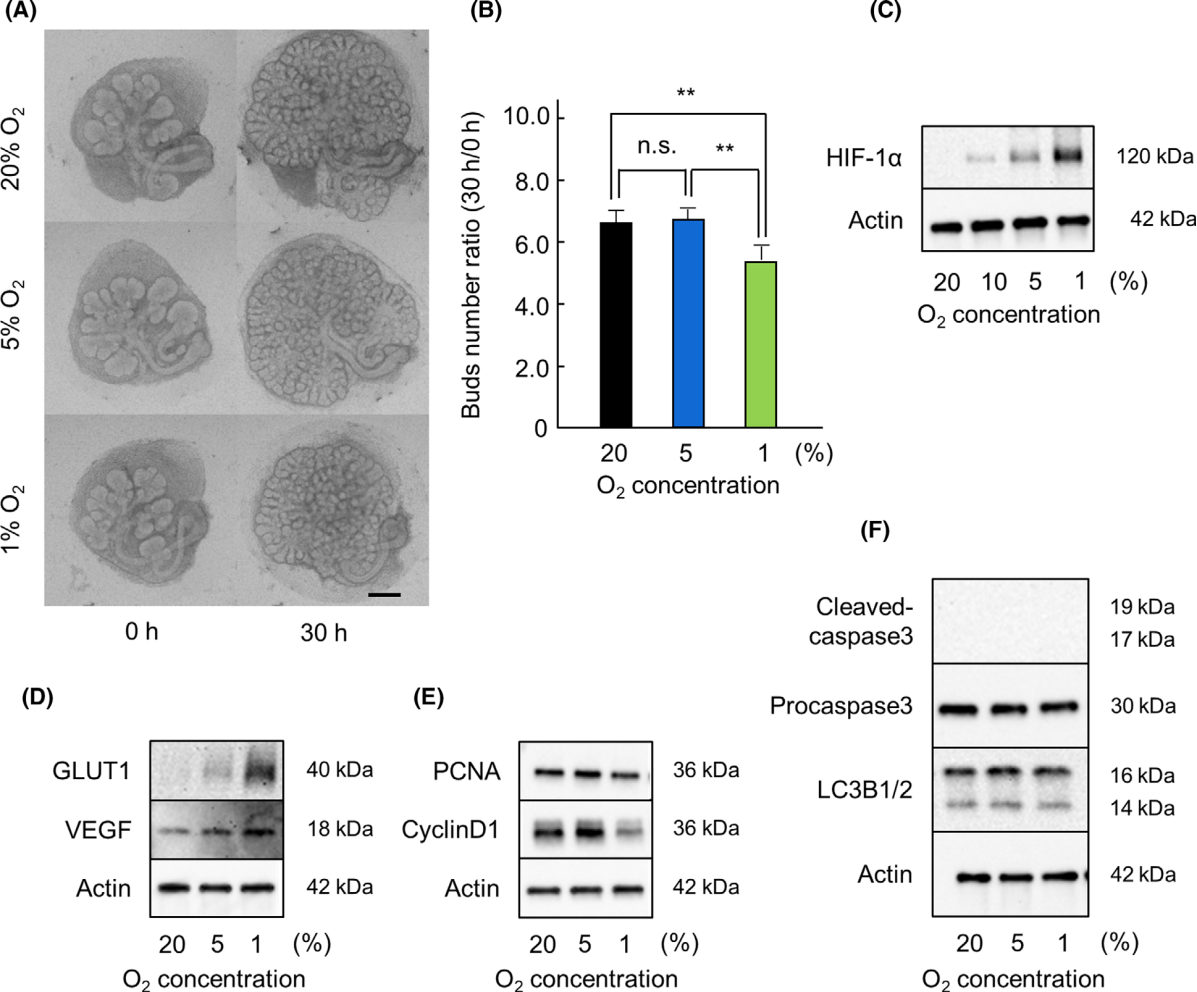
SMG branching morphogenesis in *ex vivo* organ culture under normoxic, hypoxic, and severe hypoxic conditions. Phase‐contrast images show E13.5 + 18 h cultured SMGs after 0 and 30 h of culture with DMSO under normoxic (20% O_2_), hypoxic (5% O_2_), and severe hypoxic (1% O_2_) conditions (*n* = 5) (A). The effect of hypoxic conditions was quantified by counting the number of buds per gland at 0 and 30 h (*n* = 5) (B). The expression of HIF‐1α in E13.5 + 18 h cultured SMGs after 4 h of culture with DMSO under normoxic (20% O_2_) and hypoxic (10%, 5%, 1% O_2_) conditions was analyzed by western blot analysis (C). The expression of GLUT‐1, VEGF, PCNA, Cyclin D1, Cleaved‐caspase‐3, Procaspase‐3, LC3B1/2, and actin in E13.5 + 18 h cultured SMGs after 4 h of culture with DMSO under normoxic (20% O_2_), hypoxic (5% O_2_), and severe hypoxic (1% O_2_) conditions was analyzed by western blot analysis (D, E, F). Bars represent the mean ± SEM. n.s. indicates not significant (*P* > 0.05). **P < 0.01 compared with each sample (one‐way analysis of variance (ANOVA) followed by Bonferroni’s multiple comparison test). Scale: 250 μm.

When tissues are exposed to severe hypoxic conditions, they are damaged [21, 22]. There have been no reports on the effect of severe hypoxic conditions on salivary gland development. Hence, to demonstrate the effect of severe hypoxia on salivary gland development, SMG were cultured under normoxic (20% O_2_), hypoxic (5% O_2_), and severe hypoxic (1% O_2_) conditions. Severe hypoxic conditions (1% O_2_) drastically affected bud number and development compared to the other conditions tested (Fig. 6A,B). We performed western blot analysis to detect the expression of HIF‐1α, GLUT1, and VEGF under 20%, 10%, 5%, and 1% O_2_ conditions. We found that the expression of HIF‐1α increased with decrease in the oxygen concentration (Fig. 6C). Similarly, the expression of GLUT1 and VEGF also increased as the oxygen concentration decreased. Although the expression of HIF‐1α, GLUT1, and VEGF increased under severe hypoxic (1% O_2_) conditions (Fig. 6D), the growth of SMG was poor (Fig. 6A,B). Therefore, we investigated the effect of hypoxic conditions on cell proliferation and cell death, as previous studies have reported the role of cell proliferation and cell death during salivary gland development [7, 8]. We investigated the expression of PCNA, Cyclin D1, Cleaved‐caspase‐3, Procaspase‐3, and LC3B1/2 by western blot analysis. The expression of PCNA and Cyclin D1 increased the most under 5% O_2_ condition, followed by 20% and 1% (Fig. 6E). No differences were observed in the expression of Cleaved‐caspase‐3, Procaspase‐3, and LC3B1/2 with regard to change in the oxygen concentration (Fig. 6F). These results indicated that although HIF‐1α was expressed under severe hypoxic (1% O_2_) conditions, it was not able to promote salivary gland development to the same extent as it did under hypoxic (5% O_2_) conditions.

## Discussion

During development, SMG branching morphogenesis is regulated by various factors and cytokines via many signaling cascades [8], but the role of the HIF‐1α‐related signaling pathway in salivary gland development has not been investigated. Using *ex vivo* SMG organ culture, we found that the HIF‐1α‐related signaling pathway regulates branching morphogenesis during SMG development. First, we cultured SMG under hypoxic conditions (5% O_2_) and found that the growth was not inferior to that of SMG cultured under normoxic conditions (Fig. 1A). The morphology was also similar under these two conditions, and no difference in the number of buds was noted (Fig. 1B). We confirmed that HIF‐1α was not expressed under normoxic (20% O_2_) conditions but was expressed under hypoxic (5% O_2_) conditions (Fig. 1C). Under 5% O_2_ conditions, the expression of GLUT1 and VEGF, downstream factors of HIF‐1α [2, 23], was also observed (Fig. 1D). Therefore, we hypothesized that HIF‐1α may contribute to salivary gland development under hypoxic conditions. We examined SMG development when the expression of HIF‐1α was inhibited by BAY 87‐2243 and confirmed poor growth of SMG when HIF‐1α expression was suppressed (Fig. 2A, B). Compared to untreated SMG, the number of buds decreased significantly in BAY 87‐2243‐treated SMG (Fig. 2C). This result supports the hypothesis that the presence of HIF‐1α is necessary for salivary gland development under hypoxic conditions.

Under severe hypoxic conditions (1% O_2_), SMG growth was poor despite increased expression of HIF‐1α (Fig. 6A–C). Initially, we thought that the increase in HIF‐mediated expression of GLUT1 and VEGF would help maintain salivary gland development under severe hypoxic conditions; however, SMG growth was poor. Therefore, we investigated the expression of factors involved in cell proliferation and cell cycle in SMG. In the present study, the expression of PCNA and Cyclin D1 was the highest under 5% O_2_ conditions, but decreased at oxygen concentration lower than 5% (Fig. 6E). These results indicate that under severe hypoxic conditions, the effect of decreased cell proliferation and cell cycle would overshadow the effect of HIF‐1α‐mediated increase in expression of GLUT1 and VEGF.

Next, we investigated the expression of factors involved in autophagy and apoptosis. There was no difference in autophagy and apoptosis, even with change in the oxygen concentration. Some researchers have reported two types of autophagy: (a) autophagy, which supplies nutrition, is controlled to a proper pH, and does not cause cell death, and (b) autophagy induced under severe hypoxia and metabolic stress, such as low glucose and low pH, which causes cell death [24]. Our results showed that GLUT1 and VEGF levels increased but did not cause cell death under hypoxic conditions. Thus, in this study, autophagy was potentially mediated by glucose uptake and angiogenesis in the absence of metabolic stress.

A previous study reported that hypoxic stimulation inhibits apoptosis [25]. This suppression mechanism has been suggested in various studies, some of which are HIF‐1α‐dependent, while others are HIF‐1α‐independent [26]. Another study has shown direct evidence that HIF‐1α plays a protective role against apoptosis, through a mechanism involving iron chelators [27]. Our study suggests that HIF‐1α, which increases with decrease in the oxygen concentration, prevents apoptosis. Additionally, it has been shown that the ERK1/2 pathway activated by hypoxia prevents apoptosis [28]. In our study, the activation of ERK1/2 may have prevented apoptosis as ERK1/2 is also involved in SMG development.

In summary, the results of our study suggest that the suppression of SMG growth under severe hypoxic conditions is not caused by autophagy and apoptosis but rather by the suppression of cell proliferation.

Some studies have reported that the ERK1/2 signaling pathway is essential for the development of tissues and organs, such as the respiratory tract, macrophages, and skeletal muscles, and in the regulation of homeostasis [29, 30, 31]. Our study also suggests that the ERK1/2 pathway is involved in SMG development under hypoxic conditions (Fig. 3), by activation of its downstream factors, HIF‐1α, GLUT1, and VEGF. To the best of our knowledge, this is the first study to prove that the HIF‐mediated ERK1/2 pathway is involved in salivary gland development.

This study provides the first demonstration of the effect of HIF‐1α on salivary gland development using *ex vivo* SMG. In addition, we showed that the HIF‐1α signaling pathway acts through the ERK1/2 pathway and increases the expression of its downstream substrates, such as GLUT1 and VEGF. Further investigation is necessary to understand the role of HIF‐1α in salivary gland development. We believe that conducting research using *in vivo* experiments will deepen our understanding of the role of HIF‐1α in salivary gland development.

## Conflict of interest

The authors declare that they have no conflicts of interest with the contents of this article.

## Author contributions

KT, MS, and TS conceived the experiments; KT, MS, and GN performed the experiments; KT, MS, and MW designed and performed the data analysis; KT, MS, NU, and TS co‐wrote the paper.

## Data Availability

The data that support the findings of this study are available from the corresponding author upon reasonable request.
